# A comparison of budesonide/formoterol maintenance and reliever therapy vs. conventional best practice in asthma management

**DOI:** 10.1111/j.1742-1241.2009.02185.x

**Published:** 2009-10

**Authors:** R Louis, G Joos, A Michils, G Vandenhoven

**Affiliations:** 1 Pneumology, CHU Sart TilmanLiège, Belgium; 2 Department of Respiratory Medicine University Hospital, De PintelaanGent, Belgium; 3 Chest Department, Erasme University HospitalBrussels, Belgium; 4 AstraZenecaBrussels, Belgium

## Abstract

**Objective:**

To study the effectiveness and safety of budesonide/formoterol (Symbicort®) Maintenance And Reliever Therapy (Symbicort SMART®, AstraZeneca, Södertalje, Sweden), a simplified management approach with one inhaler compared with conventional best practice (CBP) with multiple inhalers in patients with persistent asthma.

**Design:**

Open-label randomised controlled parallel group trial, 6-month treatment.

**Participants:**

A total of 908 patients ≥ 12 years of age, with persistent asthma receiving treatment with inhaled corticosteroids (ICS), either alone or in conjunction with long-acting β_2_-agonist.

**Main outcome measures:**

Time to first severe asthma exacerbation and number of severe asthma exacerbations.

**Results:**

No difference between groups was seen in time to first severe exacerbation (p = 0.75). Exacerbation rates were low in both groups. A total of 12 patients in the Symbicort SMART® group experienced a total of 14 severe asthma exacerbations, and 19 patients in the CBP group experienced a total of 25 severe asthma exacerbations (annual rate 0.07 vs. 0.13 p = 0.09). The mean daily dose of ICS expressed in BDP equivalent was significantly lower in the Symbicort SMART® group (including as-needed use) vs. in the CBP group (749 μg vs. 1059 μg; p < 0.0001). Mean scores in Asthma Control Questionnaire, 5 question version improved significantly in the SMART group compared with the CBP group (p = 0.0026). Symbicort SMART and CBP were equally well tolerated.

The mean drug cost/patient/month was significantly lower for the patients in the Symbicort SMART group compared with patients receiving CBP (51.3 € vs. 66.5 €; p < 0.0001).

**Conclusions:**

In Belgian patients, a simplified regimen using budesonide/formoterol maintenance and reliever therapy was at least as effective at improving clinical control compared with CBP with a significantly lower ICS dose and significantly lower drug costs.

**What’s known**

Although SMART strategy has proved its efficacy and safety in asthma management in controlled studies, data showing this concept may still be operating in real clinical practice are still sparse.

**What’s new**

Our study shows that, in a setting close to real clinical practice, Symbicort SMART is at least as effective as best conventional practice in controlling asthma but with a significantly lower dose of ICS and lower drug costs.

## Introduction

The primary goals of asthma treatment are to avoid severe asthma exacerbations, to control symptoms and to maintain normal lung function with the lowest effective dose of medication so that unnecessary adverse effects can be avoided. Global Initiative for Asthma (GINA) guidelines recommend a stepwise approach for asthma therapy that is based on achieving asthma control. Patients with persistent asthma, not adequately controlled by maintenance therapy with a low-to-medium dose of inhaled corticosteroids (ICS) alone, are treated with a low, and if required, a medium or high dose of ICS combined with a long-acting β2-adrenoceptor agonist (LABA), plus as-needed reliever therapy with a short-acting β2-adrenoceptor agonist (SABA). In patients not achieving target control on ICS plus LABA therapy, such as budesonide/formoterol or salmeterol/fluticasone propionate single inhaler therapy, a third controller, such as leukotriene receptor antagonist (LTRA or theophylline), should be considered as a further stepwise addition in therapy (1).

Budesonide is a potent corticosteroid that has acute effects on inflammation (2) and airway responsiveness (3). Formoterol, a rapid LABA, has been shown to improve asthma control and reduce severe exacerbations when added to budesonide (4). This rapid onset of effect has led to the development of the budesonide/formoterol combination inhaler in a single device (5) for use as both maintenance and reliever therapy (SMART) (6).

A total of six double-blind comparative clinical trials have consistently shown that the use of the combination of budesonide/formoterol with a fixed maintenance dose plus as-needed reliever therapy improved asthma control in adults and adolescents by reducing exacerbations, improving lung function and symptom control with a similar safety profile to higher doses of ICS alone (7,8) or similar or higher fixed dose ICS/LABA therapy with short-acting β2-agonist reliever therapy (9–12). The efficacy of budesonide/formoterol for maintenance and reliever therapy was also compared with any dose of salmeterol/fluticasone propionate plus salbutamol in an open-label study that for the first time provided physicians the freedom to titrate maintenance up or down in accordance with normal clinical practice with both regimen (13).

Although SMART strategy has proved its efficacy and safety in controlled studies and has also recently been included in the GINA guidelines as a valid option for both maintenance and reliever therapy (1), data showing this concept may be operating in real life are still limited. This 6-month study in outpatients aged ≥ 12 years was intended to further validate the efficacy, the safety and the cost-effectiveness of budesonide/formoterol (SMART) without changes in maintenance therapy compared with CBP in a real-life setting. The study compared the budesonide/formoterol (SMART) concept with a conventional stepwise treatment regimen in patients who presented with symptoms on ICS or patients who were symptomatic or asymptomatic on treatment with combination therapy of ICS plus LABA and/or LTRAs. Patients randomised to the Budesonide/formoterol (SMART) arm were requested to maintain only the low maintenance dose of budesonide/formoterol over the course of the trial with as-needed adjustment if symptoms occurred. In this way, the effectiveness of as-needed adjustment with Budesonide/formoterol (SMART) could be compared with physicians’ free choice of stepwise maintenance therapies defined as CBP.

## Methods

### Study design

This was a randomised, open-label, parallel-group, multicentre study. An open randomised design was necessary as an important part of the Budesonide/formoterol (SMART) concept is the use of only one inhaler. The complexity of treatment options in the CBP arm with multiple controller therapies allowed (ICS and ICS/LABAs at any dose, add-on oral leukotriene antagonist or xanthines) also excluded the possibility of blinding the comparator treatment arm. The study consisted of a 2-week run-in period followed by a 26-week randomised treatment period.

The primary objective of the study was to compare the efficacy of Budesonide/formoterol (SMART) with treatment according to CBP in adolescent and adult patients with persistent asthma. The secondary objective was to collect safety data for treatment in the two treatment groups by evaluating the incidence and types of serious adverse events (SAEs) and discontinuations because of adverse events (AEs). In addition, we compared the cost of asthma medication between the budesonide/formoterol (SMART) group and the CBP group. For this purpose, a randomised, open-label, parallel-group design was selected at 194 centres in Belgium and Luxembourg under the supervision of 305 investigators, 44 of whom were specialists and 261 general practitioners. The trial was carried out between December 2004 and June 2006.

### Participants

The inclusion/exclusion criteria were designed to select patients who presented with symptoms while on treatment with ICS, or who where symptomatic or asymptomatic on ICS and LABA therapy with or without additional controller therapy, e.g. LTRAs. The study population was therefore intended to be consistent with the use of Budesonide/formoterol (SMART) as an alternative to high dose ICS, although combination therapy was allowed in the CBP arm.

**Figure 1:**
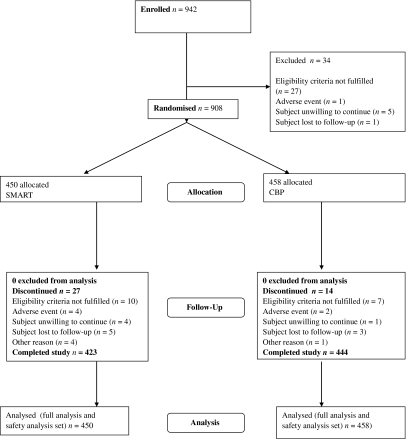
Trial profile

We enrolled out-patients of either gender aged ≥ 12 years who had been diagnosed with asthma > 3 months and who were prescribed ICS at a dose of ≥ 500 μg/day beclomethasone diproprionate (BDP) equivalent ± any other controller therapies, e.g. LABAs, LTRAs. To be eligible patients using ICS, monotherapy also needed to use ≥ 3 inhalations of as-needed medication for symptom relief during the last 7 days before enrolment. We excluded patients if they had used oral glucocorticosteroids (GCS) or if they had experienced an asthma exacerbation requiring a change in asthma treatment during the preceding 14 days (Figure 1). Participants gave written informed consent. For adolescents < 18 years old, the written informed consent form was to be signed by both the parent or legal guardian and the patient. During the run-in period, all enrolled patients were to continue on their usual asthma medications and patients who experienced an asthma exacerbation requiring change in asthma treatment during the same period were withdrawn from the study.

### Interventions

Patients started a 26-week study period receiving either SMART [budesonide/formoterol™ Turbuhaler™ (160/4.5 μg/inhalation, delivered dose)] (AstraZeneca, Södertälje, Sweden), twice daily as maintenance plus as needed in response to symptoms or CBP treatment. All investigators were encouraged to provide CBP treatment according to GINA guidelines with treatment changes allowed (i.e. any locally approved maintenance therapy excluding oral steroids) in response to increasing/decreasing symptoms or deteriorating/improving lung function in the CBP group. During treatment, visits were scheduled after 4, 13 and 26 weeks of study. Additional unscheduled visits at the initiative of physician and/or patient were allowed.

During the treatment period, SMART-treated patients were not to use more than 10 inhalations as-needed during any single day in addition to their maintenance treatment. In the unlikely event, the patient needed more as-needed medication on a single day; the investigator was to be contacted for reassessment of the patient’s condition. Patients were instructed to rinse the mouth after intake of maintenance medication (morning and evening) during the treatment period, but not after intake of as-needed medication. During the treatment period, asthma medication other than budesonide/formoterol (SMART) was only allowed for the treatment of exacerbations.

Any asthma medication including ICS and excluding SMART and/or maintenance oral steroids was considered as conventional best practice. Treatments were individually prescribed at the discretion of the investigator and within approved label for each product. During the treatment period, the CBP patient received the same instructions related to as-needed SABA medication as specified in the SMART group and as per normal practice, and patients were instructed to rinse the mouth after intake of any ICS-containing medication.

### Outcomes

The data collection process was designed to influence the patient and the physician behaviour as little as possible during the study course to maintain a real-life approach.

The primary outcome was the time to first severe asthma exacerbation defined as deterioration in asthma leading to at least hospitalisation/emergency room (or equivalent) or oral GCS treatment for at least 3 days.

Secondary outcome variables included the number of severe asthma exacerbations, the mean use of as-needed medication (reliever medication) and prescribed asthma medications. Patients completed their daily use of as-needed medication (i.e. total number of inhalations per day) in a notebook during the last 14 days before each visit during the treatment period. The patients were also instructed to document any changes in prescribed asthma medication during the study period on an ongoing basis.

To assess lung function, pre and postbronchodilator peak expiratory flow (PEF) measurements were performed with a Mini Wright™ peak flow meter (Clement Clarke, Harlow, UK) in the GP clinic at baseline and at 6-month. For patients enrolled by a lung specialist, Forced Expiratory Volume in 1-s (FEV_1_) measurements was performed in addition. At both visits, PEF and FEV_1_ measurements were performed according to standard procedures before and after administration of two inhalations of bronchodilator (BD, Ventolin™ pMDI 100 μg/inhalation, Ventolin, GSK, Evreux, France). The highest PEF and FEV_1_ value out of three attempts was recorded.

Two patient-report outcome questionnaires were used, the Asthma Control Questionnaire, 5 question version (ACQ5) (14) and the Satisfaction with Asthma Treatment Questionnaire (SATQ) (15). Both were self-administered during the study visits and completed before any other study-related procedures took place.

The overall score for the ACQ5 was the mean of the five responses. At least four out of the five questions must have been answered to provide a value. The change from baseline to the average during the treatment period (mean of Visits 3, 4 and 5) was calculated for the ACQ5 score.

The SATQ was used to measure the patient’s satisfaction with their inhaled asthma medication. The SATQ has been validated for use from the age of 18. The questionnaire includes 26 questions that are divided into four domains: effectiveness (eight questions), ease of use (seven questions), burden of asthma medication (six questions) and side effects and worries (five questions). The response options for each question are assessed on a seven-point scale. Negatively phrased items are reversed, so that a higher score indicates higher satisfaction with inhaled asthma treatment. The SATQ was administered only at baseline and at 6-month. Patients were reminded that they were scoring satisfaction with their inhaled asthma treatment and not satisfaction with other medical treatment or with the health care system.

The cost of asthma medication used over the entire study period was estimated for each patient, based on information provided in the patient notebook. The Belgian Centre of Pharmacotherapeutic IBIP website was used to calculate the minimum number of units of each medication which would have been needed to be purchased, based on product name and duration of use. Mean (with standard deviation) and median costs per patient in each treatment group were then determined.

To evaluate safety of treatment in both groups, the number and percentage of patients with SAEs and discontinuation of study treatment because of AEs were analysed. Any indications in the notebook of SAEs and/or discontinuations because of AEs needed to be discussed by the investigator with the patient.

### Statistical methods

A sample size of 500 patients per treatment group (a total of 1000 randomised patients) was required to detect a difference between the two treatment groups with 80% probability using a Log-rank test, under the assumption that, at the end of the study, 11% of the patients would have experienced a severe asthma exacerbation in one treatment group and 6% of the patients would have experienced a severe asthma exacerbation in the other treatment group.

Time to first severe asthma exacerbation was described using Kaplan–Meier curves with treatments compared using a Cox proportional hazards model with treatment as a factor. The mean number of severe asthma exacerbations per patient was compared between treatments using a Poisson regression model with treatment as factors and the time in study as an offset variable. The confidence limits and the p-value were adjusted for over dispersion. The overall ACQ5 score, overall SATQ score, use of as-needed medication, PEF, FEV_1_ and cost of asthma medication were all compared between treatments using separate analysis of variance models, with treatment as factor and the baseline visit value as a covariate. Safety data were analysed by means of descriptive statistics. A p-value < 0.05 was considered as statistically significant.

## Results

### Demographics and baseline characteristics

A total of 942 subjects were enrolled in the study and 908 subjects were randomised to either the Budesonide/formoterol SMART group or the CPB group. Overall, 867 subjects (95.5%) completed the study. The most common reason for discontinuation was the failure to meet eligibility criteria.

All patients were included in the analysis of safety and efficacy.

The demographics and key baseline characteristics of study subjects are summarised in Table 1. Demographical characteristics were comparable in the two treatment groups. The mean age of randomised subjects was 43.1 years (range: 12–87 years). Their mean daily dose of ICS during the run-in period was 579 μg (1027 μg BDP equivalent). Asthma severity at study entry was classified by investigators as mild persistent (26.8%) of the study subjects), moderate persistent (37.2%) or severe persistent (35.8%).

**Table 1:** Demographical and baseline characteristics of the full analysis/safety set

	Treatment group		
Demographical or baseline characteristic	SMART (*n* = 450)	CBP (*n* = 458)	Total (*n* = 908)
*Demographical characteristics*			
Gender (*n* and % of patients)			
Male	198 (44.0)	188 (41.0)	386 (42.5)
Female	252 (56.0)	270 (58.9)	522 (57.5)
Age (years)			
Mean	43.4	42.9	43.1
Range	12–87	13–85	12–87
Median time since diagnosis, years (range)	21.0 (0–86)	20.2 (0–78)	20.4 (0–86)
*Baseline characteristics*			
Mean ICS dose/day before randomisation (range)	570 (100–2000)	589 (320–2000)	579 (100–2000)
BDP equivalent (range)	997 (200–4000)	1058 (450–4000)	1027 (200–4000)
Mean no. of as-needed inhalations/day (range)	1.09 (0–15)	1.02 (0–11)	1.06 (0–15)
As-needed free days, % (range)	60 (0–100)	61 (0–100)	60 (0–100)

All patients eligible for inclusion in the safety set were also eligible for inclusion in the efficacy set. ICS, inhaled corticosteroids; CBP, conventional best practice.

### Efficacy

#### Severe exacerbations

**Figure 2:**
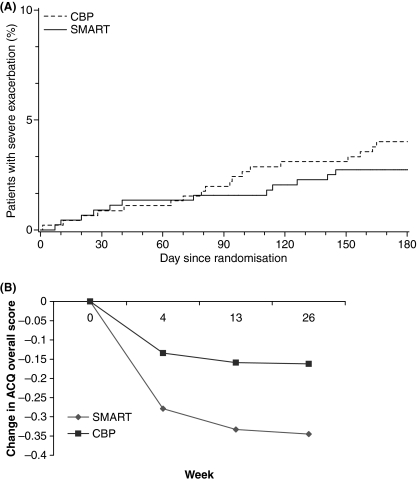
(A) Kaplan–Meier plot of time to first severe asthma exacerbation. (B) Asthma control over time assessed by Questionnaire (ACQ5). SMART = SMART; CBP = conventional best practice according to Global Initiative for Asthma (GINA) treatment guidelines

The time to first severe asthma exacerbation for each group is plotted in Figure 2A. Only 2.7% of patients who received the Budesonide/formoterol SMART regimen and 4.1% of patients treated according to conventional best practice experienced a severe asthma exacerbation during treatment (Table 2). Comparison of the time to first severe asthma exacerbation in the two treatment groups showed no difference (p = 0.75). However, the incidence in both groups was less than half that assumed when powering the study (i.e. 6% vs. 11%). Twelve patients in the Budesonide/formoterol SMART group experienced a total of 14 exacerbations, and 19 patients in the CBP group experienced a total of 25 exacerbations (annual rate including all patients, i.e. 0.074 vs. 0.13 per patient-year p = 0.09). One patient in the Budesonide/formoterol SMART group and four patients in the CBP group had emergency room treatment for a severe exacerbation. There were three asthma-related hospitalisations, two in the SMART group and one in the CBP group. The total number of days of severe asthma exacerbation was greater in the CBP group, 261 days vs. 138 days in the SMART group. Furthermore, days with the use of oral corticosteroids to treat severe exacerbations were also more limited (132 days vs. 244 days, that is a reduction of 46%) in the Budesonide/formoterol SMART group than in the CBP group.

**Table 2:** Number of patients with severe asthma exacerbations, total and by sub-criteria

Event		SMART (*n* = 450)	CBP (*n* = 458)
Severe asthma exacerbations (total)	No. of patients	12 (2.7%)	19 (4.1%)
	No. of events	14	25
	Total no. of days	138	261
Oral GCS	No. of patients	11 (2.4%)	16 (3.5%)
	No. of events	13	22
	Total no. of days	132	244
Emergency room treatment	No. of patients	1 (0.2%)	4 (0.9%)
	No. of events	1	4
	Total no. of days	1	4
Hospitalisation	No. of patients	2 (0.4%)	1 (0.2%)
	No. of events	2	1
	Total no. of days	10	15

GCS, glucocorticosteroids; CBP, conventional best practice.

#### Use of as-needed medication

The majority of patients in both groups had at least 1 day during which one or more as-needed inhalation was required (264 out of 450 patients in the Budesonide/formoterol SMART group and 291 out of 458 patients in the CBP group). Three patients in the SMART group compared with nine patients in the CBP group had at least 1 day with more than 10 as-needed inhalations.

Overall, mean daily as-needed inhalation use remained about the same in both treatment groups, and there was no statistically significant difference between groups in terms of the average number of inhalations per day and as-needed free days (Table 3).

**Table 3:** Changes from baseline in symptom control; lung function and satisfaction with asthma treatment

	Maintenance and reliever medication groups								Treatment comparison
	SMART				CPB				SMART vs. CPB
		Baseline	Treatment average/ end of treatment (visit 5)			Baseline	Treatment period average/end of treatment (visit 5)		
	*n*	Mean (range)	Mean (range)	Adjusted mean change‡	*n*	Mean (range)	Mean (range)	Adjusted mean change‡	Mean difference (95% CI, lower to upper limit), p-value
**Symptom control**									
Average number of rescue inhalations	444	1.09 (0–14.7)	0.93 (0–6.95)	−0.15	456	1.02 (0–11.4)	0.99 (0–10.4)	−0.04	−0.10 (−0.24–0.03) p = 0.1425
As-needed free days (%)	444	59.7 (0–107.1)	60.5 (0–102.4)	0.49	456	60.7 (0–107.1)	62.2 (0–114.3)	1.61	−1.12 (−4.70–2.46) p = 0.5383
ACQ5 total score	444	1.42 (0.0–4.8)	1.1 (0.0–4.2)	−0.30	449	1.31 (0.0–5.2)	1.16 (0.0–5.2)	−0.17	−0.12 (−0.20–−0.04) p = 0.0026
**Clinic lung function**									
PEF pre-BD (l/min)	450	411 (100.0–803.0)	424 .23 (85.0–748.0)	13.23	458	407 (90.0–799.0)	417.84 (60.0–763.0)	10.84	2.39 (−5.57–10.35) p = 0.5560
PEF post-BD (l/min)	450	437.88 (115.0–804.0)	447.05 (100.0–770.0)	9.17	458	435.76 (116.0–798.0)	442.95 (100.0–804.0)	7.19	1.98 (−5.36–9.31) p = 0.5970
FEV_1_ pre-BD (l)	136	2.78 (1.1–6.5)	2.78 (1.1 to 6.5)	−0.01	135	2.84 (0.8–5.6)	2.86 (0.7–6.0)	0.02	−0.03 (−0.12–0.06) p = 0.4790
FEV_1_ post-BD (l)	136	2.92 (1.2–6.3)	2.92 (0.9–6.3)	0.00	135	2.96 (0.9–5.7)	3 (0.8–6.0)	0.04	−0.04 (−0.11–0.04) p = 0.3285
**Satisfaction with asthma treatment**									
SATQ	419	4.78 (3.1–6.6)	4.81 (3.1–6.3)	0.03	419	4.78 (1.8–6.7)	4.82 (3.4–6.6)	0.05	−0.02 (−0.07–0.04) p = 0.5242

‡ ANOVA with treatment as factor (and baseline as covariate). BD, bronchodilator (Ventolin™); CI, confidence interval; ACQ5, Asthma Control Questionnaire (five questions); PEF, peak expiratory flow; FEV_1_, forced expiratory volume in 1 s; CBP, conventional best practice.

#### Peak expiratory flow and forced expiratory volume

Pre- and post-BD PEF measurements were performed at Visit 2 and Visit 5. For patients enrolled by a lung specialist, pre- and post-BD FEV_1_ measurements were also performed at these time points.

Mean PEF pre-BD increased from baseline for the subjects in the SMART group by 13.23 l/min and for the subjects in the CBP group by 10.84 l/min. The mean PEF post-BD (l/min) increased from baseline for the subjects in the SMART group by 9.17 l/min and by 7.19 l/min for subjects in the CBP group. There were no statistically significant differences between the two treatment groups for the mean pre-BD (p = 0.56) and post-BD PEF (p = 0.60) values or for the pre-BD (p = 0.48) and post-BD FEV_1_ values (p = 0.33) (Table 3).

#### Patient reported outcomes

Asthma control assessed by questionnaire improved in both groups from baseline. In the Budesonide/formoterol SMART group, the mean ACQ5 score, which assessed symptom control and activity limitation during the treatment period, decreased by −0.30 compared with −0.17 in the CBP group (p < 0.01) (Figure 2B, Table 3). Both groups showed similar overall treatment satisfaction (improvement in SATQ overall score) from enrolment to the end of the study (Table 3).

#### Prescribed controller medication

All 458 patients treated according to conventional best practice used ICS with the most commonly prescribed additional controller medication, excluding exacerbation treatment, being a long-acting β_2_-agonist in combination with ICS in a single inhaler (86%, *n* = 396). In addition, 7% of patients (*n* = 34) used a separate long-acting β_2_-agonist and 27% of patients (*n* = 125) used an LTRA as an additional controller (Table 4). Among those 458 patients, at the beginning of the study, 225 patients (49%) were treated with budesonide/formoterol. A total of 95% of the patients in the CPB group did not have modifications (step up/step down) in their treatment dosage (Table 5).

**Table 5:** Percentage of patients in CBP who stepped up or stepped down

Stepped up		Stepped down	
Categories	*n* (%)	Categories	*n* (%)
No	448 (97.82)	No	447 (97.60)
Yes	10 (2.18)	Yes	11 (2.40)

CBP, conventional best practice.

**Table 4:** Prescribed maintenance therapy in the CPB group

Prescribed maintenance medications	*n* = 458
Inhaled comb of long-acting beta-2 agonist and ICS	396 (86%)
Leukotriene receptor antagonists	125 (27%)
Separate ICS inhaler	60 (13%)
Separate long-acting beta-2 agonist inhaler	34 (7%)
Inhaled long-acting anticholinergics	19 (4%)
Xanthines	14 (3%)
Mucolytics	5 (1%)
Cromoglycate	2 (0%)

ICS, inhaled corticosteroids; CBP, conventional best practice.

The mean daily dose of inhaled steroid was significantly lower in the Budesonide/formoterol SMART (including as-needed use) group vs. the CBP group (482 vs. 589 μg/day for the actual doses, p < 0.0001 or 749 vs. 1059 μg when doses are expressed in BDP equivalents, p < 0.0001).

#### Drug costs

Estimated drug cost used for asthma during the treatment period were analysed for the two treatment groups. Mean asthma drug cost/patient/month were 51.28 € for the patients in the Budesonide/formoterol SMART group and 66.54 € for patients receiving CBP (Table 6).

**Table 6:** Direct drug costs

	Smart (€) *n* = 450	CBP (€) *n* = 458	p-value
Mean study drug cost per patient per 6 months	297	400	< 0.001
Mean study drug cost per patient per month	51	67	< 0.001
Mean study drug cost difference per patient per month	−15		< 0.001

CBP, conventional best practice.

Drug costs linked to the use of asthma medication were significantly lower (−15.26 €; p < 0.0001) for Budesonide/formoterol SMART group compared with the CBP group (Table 6).

### Safety/tolerability of the treatment

In this study, no clinically important differences between the two treatment groups were observed with regard to the overall pattern of reported SAEs and withdrawal because of AEs. Both Budesonide/formoterol SMART and CBP regimens were well tolerated and no new or unexpected safety concerns were identified. A total of 23 SAEs were reported during treatment, 11 in the Budesonide/formoterol SMART group and 12 in the CBP group. Two patients in the Budesonide/formoterol SMART group died during the course of the study, one by suicide, the other following myocardial infarction. None of the reported SAEs was considered to be related to treatment by the investigator.

Adverse events led to discontinuation in two patients (0.4%) in the SMART group and twp patients (0.4%) in the CBP group. Only one subject discontinued for a treatment-related event (sore throat in a patient in the CBP group).

## Discussion

The study conducted in Belgium and Luxembourg confirmed that Budesonide/formoterol SMART (one inhalation bid as maintenance treatment plus inhalations as needed in response to symptoms) is an effective therapy for asthma control compared with physicians’ choice of CBP in a real-life setting.

Study procedures were aimed to minimally influence patient’s behaviour and the pattern of the contacts with their physician. Given the complexity of treatment options in the conventional best practice arm, an open-label study design was chosen. This allowed the effectiveness of the single inhaler regimen without a separate as-needed inhaler, to be investigated without the need for separate reliever therapy as used in all previous double-blind studies. In the conventional best practice arm changes of the maintenance dose and other treatment, changes were permitted following any scheduled or unscheduled contact with the physician, but the same low maintenance dose of Budesonide/formoterol was used throughout in the SMART arm, this was not associated with any evidence of deteriorated asthma control or raised exacerbation and use of reliever therapy.

The incidence and severity of severe asthma exacerbations were similar for the two treatment arms, but the overall incidence was far lower than assumed when powering the study. However, the total number of reported severe asthma exacerbations (14 vs. 25, at a per anum rate per patient of 0.074 vs. 0.13, respectively) and the total number of days of severe asthma exacerbation (138 days vs. 261 days, respectively) tended to be lower in the Budesonide/formoterol SMART group compared with the CBP group.

In a previous open study comparing Budesonide/formoterol SMART with titration of maintenance treatment using salmeterol/fluticasone propionate (13), Budesonide/formoterol SMART significantly reduced severe exacerbations, but the overall incidence of events was substantially higher than in this study. In our study, unlike in the study by Vogelmeier et al. (13), patients with stable control and no history of exacerbations were allowed to enrol, and the maintenance treatment in the comparator arm could be freely changed with other controller drugs permitted, e.g. LTRAs. However, changes in maintenance treatment in the Budesonide/formoterol SMART arm or addition of other controller therapy were not allowed. The lack of significant differences between Budesonide/formoterol SMART and CBP with respect to time to severe exacerbation and exacerbation rates in this study and in other real-life studies (16,17) is therefore likely to reflect a lack of statistical power. This is linked to the low incidence of events in the CBP arm because of the inclusion of well-controlled patients and the added flexibility of treatment with a free choice of multiple maintenance therapies or the addition of formoterol as reliever therapy to the CBP arm, which is also effective in reducing exacerbation risk (18). This study has, however, confirmed that with no adjustment in maintenance ICS/LABA or add-on therapy the SMART approach had similar or better efficacy compared with multiple controller therapies and higher doses of inhaled steroid used during CBP. The most commonly prescribed asthma medications, excluding exacerbation treatment, in the 458 patients in the CBP arm were a combination treatment of an ICS and long-acting β_2_-agonist (86%) in a single inhaler with 7% also using LABA via separate inhaler as maintenance or reliever therapy. In addition, 27% of patients in the CBP arm used LTRAs such that approximately 20% of patients used of at least three controller medications. Mean as-needed inhalation was similar in both treatment groups. However, the Budesonide/formoterol SMART group had lower overall inhaled corticosteroid load. The daily ICS dose was reduced significantly in the Budesonide/formoterol SMART arm vs. the CBP arm by around 300 mcg/day (BDP equivalents). Furthermore days with the use of oral corticosteroids to treat severe exacerbations were also reduced by 46% in the Budesonide/formoterol SMART group compared with the CBP group.

The patient’s perception of asthma control, weekly symptoms and activity limitation assessed by a validated asthma control questionnaire (ACQ5) was improved to a greater extent over the study period with Budesonide/formoterol SMART than with CBP, although the two groups of patients were equally satisfied with their treatment at the end of the study. Of note is the fact that more than 95% of the patients did not change the maintenance treatment in the CBP group despite the fact that ACQ5 was > 0.75 for most of the patients. However this trial was undertaken 1 year before the publication of the GINA that recommended the use of ACQ in clinical practice to assess asthma control and adjust the treatment accordingly. That might explain the relative passivity of the doctors in adjusting the dose of maintenance treatment based on ACQ value.

In this study, overall Budesonide/formoterol SMART treatment was well tolerated as CBP and resulted in similar or better asthma control with less intake of ICS compared with CBP. A *post hoc* analysis also showed that drug costs linked to asthma medication used over the entire study period were significantly lower by approximately 25% for the Budesonide/formoterol SMART group. This is only relevant if we actually calculate the direct healthcare cost and not the drug cost alone. In this study, costs linked to hospitalisation were very low because of a few numbers of exacerbations. However, as hospitalisation days were lower in SMART group than in CBP group, taking into account this parameter would even strengthen the cost difference between the two strategies. This cost-effectiveness of the SMART strategy in Belgium/Luxembourg is keeping with the results of a larger study conducted according to a double-blind design (19).

In conclusion, in this real-life study performed in a wide range of asthma patients suitable for combination therapy, we found Budesonide/formoterol SMART to have similar or better efficacy and safety profile as physicians free-choice of guideline based maintenance therapy (CBP) in maintaining or improving asthma control and reducing exacerbations. Budesonide/formoterol SMART also reduced corticosteroid use, the need for multiple controller therapies and treatment costs.
